# Breathing new insights into the role of mutant p53 in lung cancer

**DOI:** 10.1038/s41388-024-03219-6

**Published:** 2024-11-20

**Authors:** Tianwei Chen, Lauren M. Ashwood, Olga Kondrashova, Andreas Strasser, Gemma Kelly, Kate D. Sutherland

**Affiliations:** 1 Walter and Eliza Hall Institute of Medical Research, Parkville, VIC Australia; 2 Department of Medical Biology, The University of Melbourne, Parkville, VIC Australia; 3 QIMR Berghofer Medical Research Institute, Herston, QLD Australia; 4 The University of Queensland, Brisbane, QLD Australia

**Keywords:** Non-small-cell lung cancer, Small-cell lung cancer, Apoptosis

## Abstract

The tumour suppressor gene *p53* is one of the most frequently mutated genes in lung cancer and these defects are associated with poor prognosis, albeit some debate exists in the lung cancer field. Despite extensive research, the exact mechanisms by which mutant p53 proteins promote the development and sustained expansion of cancer remain unclear. This review will discuss the cellular responses controlled by p53 that contribute to tumour suppression, *p53* mutant lung cancer mouse models and characterisation of *p53* mutant lung cancer. Furthermore, we discuss potential approaches of targeting mutant p53 for the treatment of lung cancer.

## The tumour suppressor p53

**Fig. 1:**
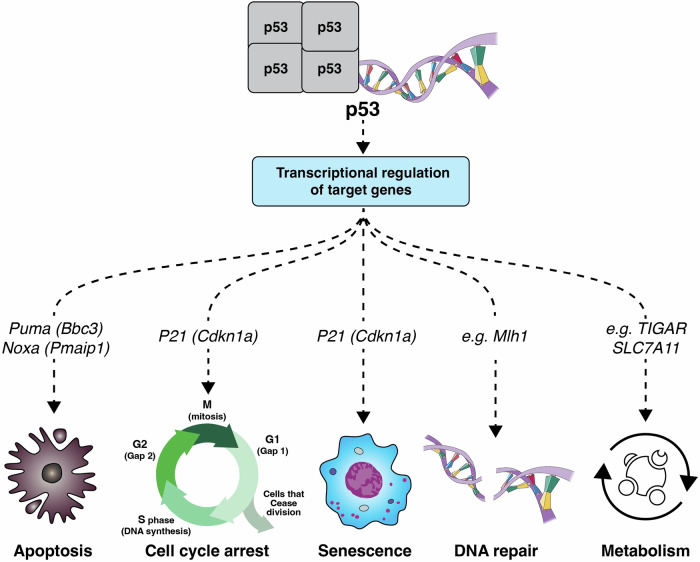
Cellular processes regulated by p53. p53 regulates diverse cellular processes via the transcriptional regulation of target genes. Some cellular processes that contribute to the tumour suppressive function of p53 include apoptosis, cell cycle arrest, cell senescence, DNA repair and cell metabolism. Some genes essential for carrying out these processes are indicated.

The *p53* gene (generic name used here, also known as *TP53* in humans and *Trp53* in mouse) encodes the tumour suppressor protein p53 (TP53/TRP53), a transcription factor that can regulate diverse cellular processes, including apoptotic cell death, adaptation of cellular metabolism, cell cycle arrest, cellular senescence and DNA damage repair [1] (Fig. 1). In unstressed cells, p53 proteins levels are kept low, since it is ubiquitinated by the E3 ligase mouse double minute 2 (MDM2) (called HDM2 in humans), thereby targeting it for proteasomal degradation. Stress conditions, such as DNA damage, hypoxia, nutrient deprivation or oncogene activation, lead to the inhibition of MDM2 and/or direct modification of p53, resulting in the stabilisation and thus activation of the p53 protein. p53 proteins then bind as homo-tetramers to the promoter regions of ~500 direct target genes and induce their expression to activate apoptosis, cell cycle arrest, DNA repair and other cellular processes [2].

Although p53 has been widely recognised as an important tumour suppressor, the exact mechanisms by which this transcription factor prevents tumour development is not fully understood. Initially, it was thought that the induction of apoptosis was the principal mechanism for tumour suppression by p53. However, numerous studies have shown that additional cellular processes must also be involved. Here, the major cellular processes that can be induced by p53 and how they contribute to tumour suppression will be discussed individually.

## Apoptosis

Apoptosis is a form of programmed cell death that is important for embryonic development, and the maintenance of tissue homoeostasis by removing no longer needed or potentially dangerous (e.g. infected or early neoplastic) cells [3–5]. Apoptosis can occur through two distinct yet converging pathways, the intrinsic (aka mitochondrial, stress-induced or BCL-2 regulated, BAX/BAK-dependent) or extrinsic (aka death receptor-induced) pathways [6]. The intrinsic apoptotic pathway is regulated by the proteins of the B cell lymphoma 2 (BCL-2) family [7]. The BCL-2 family of proteins are divided into three subgroups: (1) the pro-apoptotic BH3-only proteins, such as Bcl-2 Interacting Mediator of cell death (BIM), the p53 upregulated modulator of apoptosis (PUMA) and phorbol-12-myristate-13-acetate-induced protein 1 (NOXA), which are initiators of apoptosis; (2) the pro-survival proteins, such as BCL-2, BCL-XL and MCL-1, which are the guardians that inhibit apoptosis; and (3) the pro-apoptotic effectors of apoptosis, BAX, BAK (and BOK), with the first two inhibited by the pro-survival BCL-2 proteins in healthy cells [6]. The genes that encode the BH3-only proteins, PUMA and NOXA, have been identified as direct target genes of p53, whereas the gene that encodes the BH3-only protein BIM can be indirectly induced by p53 [8, 9]. Upon certain cellular stresses, p53 is activated and then increases the expression of PUMA, NOXA and BIM, which inhibit the pro-survival BCL-2 proteins, leading to the activation of the effectors of apoptosis, BAX and BAK. It has been reported that BAX and BAK can also be directly activated by some BH3-only proteins [7]. When activated, BAX and BAK oligomerise and cause mitochondrial outer membrane permeabilisation (MOMP). This leads to the release of apoptogenic factors, such as cytochrome c and SMAC/DIABLO into the cytoplasm. Cytochrome c binds to apoptotic peptidase-activating factor 1 (APAF-1) and this adaptor platform induces activation of the initiator caspase, caspase-9. Caspase-9 then proteolytically activates the downstream effector caspases (i.e. caspases-3, (-6) and -7) and this caspase cascade causes the ordered dismantling of the dying cells [7]. The extrinsic apoptotic pathway is triggered by the stimulation of death receptors, such as FAS, by their ligands, such as FASL, on the plasma membrane. This activates the initiator caspase, caspase-8, through the adaptor protein FADD. Activated caspase-8 can trigger apoptosis either directly by proteolytically activating the downstream effector caspases, caspases-3, (-6) and -7, however can also proteolytically activate the BH3-only protein BID, thereby increasing effector caspase activation by engaging the intrinsic apoptotic pathway (see above) [10]. p53 is thought to also be involved in the regulation of the death receptor apoptotic pathway by upregulating target genes, such as those for FAS [11, 12], Death Receptor 5 (DR5) [13], TNF receptor-associated protein 4 (TRAF4) [14] and SUSD6 (DRAGO; TMPS; KIAA0247) [15, 16]. Of note, cells lacking FAS (e.g. from *lpr* mutant mice) or cells overall deficient in death receptor-induced apoptosis (e.g. lacking FADD function) are resistant to apoptosis induced by activation of p53 by γ-radiation or DNA damage-inducing drugs [17]. Therefore, the upregulation of these genes by p53 possibly serves to sensitise cells to apoptosis by cytotoxic T cells and natural killer (NK) cells, which can express the ligands for death receptors.

The ability of p53 to induce apoptosis was first revealed in a study using a myeloid leukaemia cell line in which expression of wildtype (WT) p53 was induced [18]. Subsequently, using gene knockout mice, it was shown that p53 is essential for the induction of apoptosis triggered by agents that cause DNA damage, including γ-radiation and certain chemotherapeutic drugs, such as etoposide or cisplatin [19–21]. Later work demonstrated that the combined absence of the p53 target genes encoding the pro-apoptotic BH3-only proteins PUMA and NOXA (and to a lesser extent loss of PUMA alone) rendered lymphoid cells as resistant to DNA damage-inducing agents as loss of p53 itself [8, 9, 22]. This demonstrated that transcriptional induction of PUMA and NOXA accounts for all p53-induced apoptosis. As soon as p53 was shown to be able to induce apoptosis it was predicted that this is one of, if not the sole, mechanism by which p53 prevents tumorigenesis. Consistent with this notion, loss of PUMA accelerates c-MYC over-expression induced lymphoma development in mice [23]. However, even combined loss of PUMA and NOXA accelerated c-MYC induced lymphomagenesis to a considerably lesser extent than loss of only a single allele of *p53* (*p53*^*+/−*^) [23]. This demonstrates that p53 must suppress tumour development not only through induction of apoptosis, but also through activation of additional processes.

## Cell cycle arrest and cell senescence

Cell cycle arrest and cell senescence, the latter a process of irreversible cell cycle arrest, are protective mechanisms for maintaining tissue homoeostasis, and can function as complementary mechanisms to programmed cell death for tumour suppression [24]. Cell cycle arrest and cell senescence can be triggered by shortening of telomeres, a feature of aged cells, as well as in response to various environmental and cellular stresses, such as nutrient deprivation, DNA damage and oncogene expression. One of the most extensively studied pathways of induction of cell cycle arrest and cell senescence is through the direct transcriptional induction of the cyclin-dependent kinase inhibitor p21 by p53, resulting in arrest at the G1/S boundary [25]. In addition to proliferation arrest, senescent cells also exhibit alterations to their morphology (increased flattening) and metabolism with the activation of the so-called senescence-associated secretory phenotype (SASP), whereby the cells secrete cytokines, chemokines and proteases, that have profound effects on neighbouring cells in the tumour microenvironment (TME) [26–28].

p53 has been shown to be essential for oncogene-induced senescence [29]. For example, the expression of mutant *Rat sarcoma virus (*RAS) in mouse embryonic fibroblasts results in permanent G1 cell cycle arrest accompanied by a significant increase in the level of p53 and the expression of many of its target genes, including p21. The loss or mutation of *p53* prevents mutant RAS induced cell cycle arrest and senescence, thereby promoting the malignant transformation of these cells [30]. However, in recent years some studies have provided evidence that senescent cells can under certain conditions also promote tumorigenesis [31, 32]. The depletion of senescent cells in aging mice delayed age-related morbidity and decreased the incidence of tumorigenesis and cancer-related death [33]. This is thought to be due to the removal of the SASP that may promote tumorigenesis by inducing chronic inflammation, a known driver of tumorigenesis [27, 32, 34]. Moreover, it has been reported that under certain conditions senescent cancer cells are capable of exiting from their growth arrested state, thereby re-starting tumour expansion [35]. These features of senescent cells are identified as the emerging hallmarks and enabling characteristics of cancer [24].

Although p53 induced apoptosis via PUMA and NOXA and cell senescence via p21 play critical roles in preventing cells from malignant transformation, these two mechanisms are dispensable for p53-mediated suppression of spontaneous tumour development. Remarkably, none of *Puma*^*−/−*^, *Noxa*^−*/−*^, *Puma*^−*/−*^*;Noxa*^*−/−*^ or even *Puma*^−^^*/−*^*;Noxa*^−^^*/−*^*;p21*^*−/−*^ mice spontaneously developed tumours even when aged to >450 days. In contrast, *p53*^*−/−*^ mice (on a C57BL/6 background) develop tumours spontaneously with a 100% incidence within 280 days [36, 37]. Similarly, mice with *p53* mutations that impair the transcriptional activation of *p21*, *Puma* and *Noxa*, but retain the ability to induce the transcription of certain other p53 target genes, also do not develop tumours spontaneously [38]. Collectively, these findings indicate that p53-mediated tumour suppression is a complex process and that p53 target genes other than *Puma*, *Noxa* and *p21* and cellular processes in addition to apoptosis and cell cycle arrest/cell senescence must also be involved.

## DNA damage repair

DNA damage repair is a critical cellular response to maintain genomic integrity after the exposure to endogenous or exogenous DNA damaging agents [39]. Failure of DNA damage repair can result in genomic instability and mutations, and this is also identified as a hallmark of cancer [5]. Several DNA damage response processes operate in cells to prevent propagation of DNA lesions. For example, nucleotide excision repair (NER) removes adducts of DNA lesions commonly caused by UV irradiation and is one of the major mechanisms that protect cells from neoplasm, mutagenesis and cytotoxicity [40]. On the other hand, base excision repair (BER) corrects oxidative modifications of the DNA bases [41]. DNA double stranded breaks (DSB) are typically induced by ionising radiation or spontaneously during DNA replication. These lesions are repaired through homologous recombination (HR) or non-homologous end joining (NHEJ). Mismatch repair (MMR) rectifies nucleotides that have been incorrectly inserted into DNA during the replication process [42].

Many studies have provided evidence that p53 plays an important role in maintaining genome stability and suppressing tumorigenesis by activating a variety of DNA damage repair pathways [42]. The earliest connection between p53 and NER was demonstrated by Smith et al., where they found that human cells in which normal p53 function was disrupted by either the human papillomavirus E6 oncoprotein or expression of a transgene encoding a dominant-negative mutant of p53 showed significantly reduced tolerance to UV irradiation [43]. In BER, various interactions were described between p53 and APE1/Ref-1, the apurinic and apyrimidinic (AP) endonucleases that are essential for the removal of damaged bases and subsequently the repair of the AP sites [44, 45]. It was reported that APE1/Ref-1 can promote the tetramerization of p53 [46] and regulate its trans-activation and pro-apoptotic functions [44]. Interestingly, p53 was found to directly supress the transcription of *APE1*. This function is thought to be related to the tumour suppressive role of p53, as the downregulation of BER can bias the cellular response towards apoptosis in cells with highly damaged DNA [47]. Functions of p53 in MMR are mainly exerted through its transcriptional induction of the gene encoding the MMR core component MSH2. However, MSH2 is also found to function in NER, BER and HR. Therefore, p53-dependent induction of MSH2 expression may also be involved in other DNA damage repair pathways [42]. Additionally, p53 has been shown to activate the HR pathway by both direct and indirect interactions with RAD51 [48–52]. Other studies have shown that p53 can also mediate HR by interacting with other proteins, such as the ATM/ATR checkpoint kinases [53]. The role of p53 in NHEJ is less well studied compared to those in HR. It is known that p53 can promote NHEJ by interacting with components of the NHEJ pathway, such as XRCC4 and DNA ligase IV [54–56]. However, the molecular mechanisms of these interactions remain largely unclear. Recently, a *p53*-dependent gene *ring finger protein 144b (Rnf144b*) has been found to be involved in the repair of DNA DSBs and is shown to have a pivotal tumour suppressive function, particularly in lung adenocarcinoma [57]. In addition, an in vivo shRNA library screen and validation using CRISPR induced gene deletion revealed that the loss of several p53-regulated DNA repair genes can markedly accelerate c-MYC-driven lymphomagenesis and the removal of *Mlh1*, a key gene involved in MMR, can even on its own promote spontaneous tumour development [58]. Overall, these findings demonstrate that coordination of DNA damage repair contributes substantially to p53-mediated tumour suppression.

## Adaptation of cell metabolism

Recent studies have demonstrated that p53 may also prevent tumour development through the regulation of cell metabolism. The emerging role of p53 in metabolic rewiring in cancer has been comprehensively explored in several recent reviews [59–61], and we discuss this topic here only relatively briefly. p53 has been reported to play important roles in the control of mitochondrial oxidative phosphorylation (OXPHOS), a cellular process that produces adenosine triphosphate (ATP). p53 enhances OXPHOS by inducing the expression of cytochrome c oxidase 2 (SCO2). In addition, p53 is known to negatively regulate cellular glycolysis by directly or indirectly inhibiting the expression of glucose transporters (GLUTs) or the translocation of GLUT1 to the plasma membrane, leading to decreased glucose uptake [62–64]. p53 can also influence glucose metabolism by direct or indirect regulation of the expression of genes encoding enzymes involved in this process, such as p53-induced glycolysis and apoptosis regulator (TIGAR) [65]. The activation of TIGAR leads to a decrease in fructose-2,6-bisphosphate, which subsequently halts glycolysis. However, the absence of *Tigar* does not promote tumour development, indicating that it does not exert a pivotal role in p53-mediated tumour suppression [66].

p53 can also negatively regulate other cellular pathways involved in glycolysis, such as the PI3K/AKT signalling pathway to suppress glycolysis in cells [67–69]. Tumour cells often undergo metabolic reprogramming to support increased cell growth and proliferation. Most tumours experience a significant increase in glycolysis due to insufficient OXPHOS, which was first discovered by Warburg et al. and was named as the Warburg effect [70]. Recent studies suggest that mutations or loss of p53 in tumours could be a leading factor that results in the Warburg effect [71]. Reactive oxygen species (ROS) are by-products of OXPHOS generated when cells sustain genotoxic damage or mitochondrial stress and they are known to contribute to tissue damage and tumorigenesis [71].

Furthermore, p53 has been shown to regulate cell metabolism through crosstalk with various cellular pathways, such as the mammalian target of rapamycin (mTOR) and AMP-activated protein kinase (AMPK) pathways [72]. The mTOR pathway can be activated by high levels of nutrients and energy, such as a high ATP/ADP ratio, growth factors and oxygen, which promote anabolism and inhibit catabolism [72, 73]. Conversely, a low energy state (i.e. a low ATP/ADP ratio) activates AMPK, which in turn promotes catabolism and inhibits anabolism [72, 74]. AMPK and mTOR are key regulators of autophagy, which is a self-eating process to degrade proteins, organelles and membranes, thereby recycling macromolecules for production of energy in response to cellular stress [75]. Several studies have suggested that WT p53 can induce autophagy in cancer cells via the activation of AMPK and the suppression of mTOR by transcriptional induction of the genes encoding phosphatase and tensin homologue (PTEN) and tuberous sclerosis 1 (TSC1) [76–79], whereas autophagy is inhibited in p53 mutant cancer cells [80, 81]. Moreover, cancer cells expressing mutant p53 exhibit increased sensitivity to mTOR inhibition compared to cancer cells with WT p53 [81]. This supports the importance of p53 in regulating the mTOR signalling pathway to suppress tumour development. Collectively, these findings suggest critical, albeit not yet well understood, roles of p53 in metabolic regulation and the prevention of tumorigenesis.

## *p53* mutant cancers

**Fig. 2:**
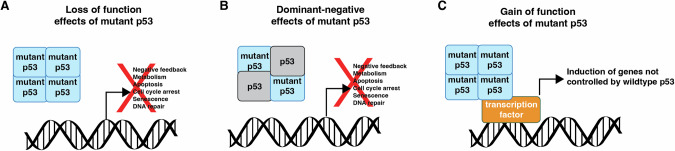
Consequences of point mutation for p53 function. WT p53 proteins bind to DNA and activate the downstream signalling pathways. **A** Loss of function (LOF) effects of mutant p53 abrogate normal p53-mediated cellular processes. **B** Dominant negative effects (DNE) of mutant p53 are a consequence of mixed tetramers containing both WT and mutant p53 proteins, which are significantly less efficient at activating the WT p53 target genes compared to tetramers containing only WT p53. **C** Gain of function (GOF) effects of mutant p53 proteins are thought to be exerted by binding to and modulating the functions of transcription factors not bound and impacted by WT p53.

*p53* mutations occur in ~50% of human cancers and are commonly associated with poor therapy responses and poor patient outcomes [82]. People with Li-Fraumeni syndrome that inherit one mutant *p53* allele and one WT *p53* allele have an ~50% likelihood of developing cancer by the age of 35 and a ~90% risk of developing cancer throughout their lifetime [83]. Although over 2000 different mutations in the *p53* gene have been reported in human cancers [84], most mutations occur at six hotspot amino acid residues in the DNA-binding domain (R175, G245, R248, R249, R273 and R282) [85]. Mutations have also been found in other domains of p53, but their effects on tumorigenesis are less well known [86]. Moreover, the mechanisms of the selection for hotspot mutant *p53* in cancers still remains unclear [87]. Interestingly, the frequency of different *p53* mutations varies dramatically between cancer types [88]. Two possible explanations for this variability are that (i) specific mutations can be preferentially enriched due to differences in mutational aetiologies (e.g. exposure to different carcinogens), and (ii) individual hotspot mutations may exhibit distinct tumorigenic potential in different tissues [89]. Mutant p53 is proposed to promote tumour development in three ways (Fig. 2). (1) Loss of function (LOF) effects abrogate normal p53-mediated cellular processes, leading to defects in cell cycle arrest, evasion of apoptosis and genomic instability [90]. (2) Dominant negative effects (DNE) are thought to result from the formation of mixed tetramers containing both WT p53 and mutant p53 proteins. In this setting, such mixed tetramers are significantly less efficient at activating target genes compared to WT p53 homo-tetramers [1]. The DNE of mutant p53 can be critical during early stages of tumour development when cells express both the WT and mutant p53 alleles [85], as at diagnosis most mutant p53 expressing cancers have lost the WT *p53* allele (loss of heterozygosity). (3) The reported gain of function (GOF) effects of mutant p53 proteins are thought to be exerted by binding to and modulating the functions of certain transcription factors (e.g. erythroblast transformation specific proteins) that are not bound and impacted by WT p53 [82, 88]. Indeed, enforced expression of mutant p53 in p53-deficient mouse or human cell lines has been reported to lead to new phenotypes [91], supporting the GOF effects of mutant p53. Moreover, previous studies showed that different *p53* mutations may have distinct oncogenic properties, and these mutations may promote tumour development beyond the LOF effect. For example, several mouse models have shown that certain mutant p53 proteins are able to enhance the invasion and motility of cancer cells compared to what is seen in p53-deficient mice [92–95]. In addition, Li-Fraumeni patients with certain hotspot *p53* mutations, such as R248Q, show earlier onset of malignant disease compared with patients that harbour mutations that cause a loss of p53 protein [96]. This may, however, also be ascribed to the DNE of R248Q mutant p53 protein. The relative importance of the LOF, DNE and GOF effects of mutant p53 in the initiation, sustained growth and metastasis of cancer is still hotly debated, as is how the different *p53* mutations affect these tumorigenic processes. Adding to the complexity, *p53* mutations have been reported to occur at different stages of malignant transformation [97]. It is therefore possible that mutant p53 proteins may contribute differently to tumorigenesis depending on the timing of the acquisition of the mutation and the context of co-existing oncogenic drivers. This still requires substantial further investigation.

## Lung cancer

**Fig. 3:**
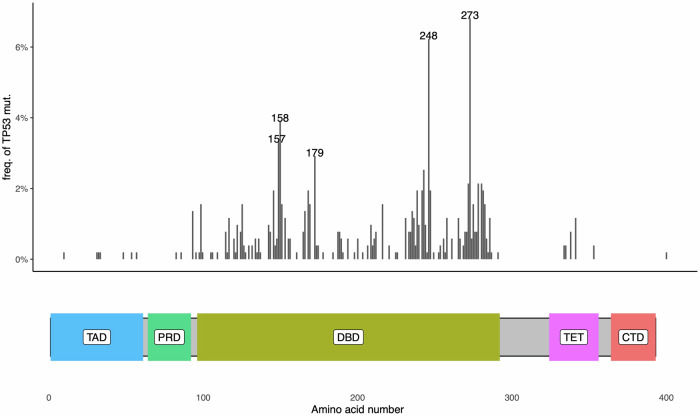
*p53* missense mutations in human LUAD. Frequencies of p53 mutants in LUAD patients with *p53* missense mutations (*N* = 514) were obtained from the GENIE BPC NSCLC v2.0 cohort [219], with domains of the p53 protein displayed below [220]. The protein schematic was generated using the drawProteins package [221]. TAD transactivation domain, PRD proline-rich domain, DBD DNA-binding domain, TET tetramerisation domain, CTD C-terminal regulatory domain.
  Conventional therapies for NSCLC include surgery, radiotherapy and chemotherapy, either alone or in combination [114]. Surgical resection is recommended for patients with early-stage NSCLC without other morbidities [115]. Platinum-based chemotherapy (e.g. cisplatin) in combination with other cytotoxic agents are standard regimens for patients with advanced lung cancer and they have been shown to significantly improve patient survival. However, these treatments have substantial toxicity and the recurrence rate of malignant disease is still high [116]. Immunotherapy is a new modality for cancer therapy. Immune checkpoint blockade (ICB) agents, such as antibodies that inhibit PD-(L)1 or CTLA-4, have been used with success in the treatment of many cancers, including NSCLC [117]. This has demonstrated higher clinical efficacy compared with conventional therapies in NSCLC [117]. However, only a small subset of patients experience durable anti-tumour responses upon anti-PD-1 treatment, and accurate biomarkers predictive of response to such therapies are yet to be defined [118]. Targeted therapy has transformed the therapeutic landscape of lung cancer over the past few decades. The identification of targetable genetic alterations has allowed for individualised therapies, and this has significantly improved the response rate and progression-free survival. Current Food and Drug Administration (FDA)-approved targeted therapies include tyrosine kinase inhibitors inhibiting EGFR, ALK, ROS1, RET, BRAF V600E, MET Exon14 or NTRK [114]. For decades, KRAS was considered an elusive target for direct inhibition. Recently, however, sotorasib (AMG 510), a KRAS^G12C^ selective inhibitor has been approved by the FDA as a second-line treatment for patients with advanced *KRAS*^*G12C*^ mutant NSCLC. This treatment has demonstrated remarkable efficacy with substantial response and disease control rates [119, 120]. A second KRAS^G12C^ inhibitor, adagrasib (MRTX849), has also produced promising outcomes in clinical trials [121, 122] and several other KRAS^G12C^ inhibitors have entered clinical studies [123]. However, there are currently no approved inhibitors of other KRAS mutants. Recent studies have reported that two drugs, MRTX1133 and BI-KRASG12D1-3, are able to selectively inhibit the viability of *KRAS*^*G12D*^ mutant tumour cells in clinical trials [123]. In addition, several indirect pan-KRAS inhibitors are currently under investigation [124, 125].
ii. Small cell lung cancer
  SCLC is a clinically and histologically distinct subgroup of lung cancer and is identified as one of the high-grade lung cancers of neuroendocrine origin [100]. This is a highly aggressive disease due to its high growth rate, early metastasis and poor prognosis. As a result, more than two-thirds of patients present with metastatic SCLC at the time of diagnosis [126]. Over 90% of SCLC patients are current or past smokers, and the risk of SCLC increases with smoking duration and intensity [127]. Unlike NSCLC, the classification of SCLC is not defined by its somatic mutations, but according to the expression of lineage-specific transcription factors. In seminal findings by Rudin et al., SCLC subtypes were defined by the expression of four transcription factors: achaete-scute homologue 1 (ASCL1), neurogenic differentiation factor 1 (NEUROD1), yes-associated protein 1 (YAP1) and POU class 2 homeobox 3 (POU2F3) [128]. Notably, SCLC tumour cells expressing ASCL1 and NEUROD1 exhibit neuroendocrine features whereas those expressing YAP1 and POU2F3 exhibit a neuroendocrine low phenotype [129–131]. However, there is increasing evidence that YAP1 does not define a distinct subtype of SCLC. Instead, a group of SCLC tumours was identified with low expression of ASCL1, NEUROD1 and POU2F3. This subtype shows a unique gene expression signature involving human leucocyte antigens and many immune checkpoint regulators, including PD-L1 and CTLA-4. Therefore, this subtype is called SCLC-Inflamed and has been found to show greater response to ICB therapy compared with other subtypes of SCLC [132]. To date, no definitive positive selection marker exist to identify this rarer subtype of SCLC tumours [132].
  Genomic profiling of SCLC has revealed the nearly universal loss of the tumour suppressor genes *p53* (75–90%) [133, 134] and *retinoblastoma* (*RB1*) (almost 100%) [135]. Apart from these shared features, other genetic alterations, such as the loss of *PTEN*, NOTCH receptors and the CREB binding protein, are also observed and the importance of these defects has been functionally validated in vivo and in vitro [126]. Epigenetic and transcriptomic analyses have identified the amplification of the *MYC* family proto-oncogenes (*MYC*, *MYCL* and *MYCN*) [136], with *MYCL* being associated with the ASCL1-subtype and *MYC* being associated with other subtypes [126]. In addition, in the *Myc*^*T58A*^*, NEUROD1*^*high*^ mouse model of SCLC, the stabilisation of the mutant MYC protein was shown to promote cell transformation and the proliferation of lung cancer cells [128].
  The standard treatment for patients with limited-stage SCLC is concurrent radiation and chemotherapy which typically includes platinum-based agents in combination with etoposide [137]. About 20% patients will achieve long-term control of malignant disease. However, disease relapse occurs in most patients, probably due to the selection for surviving treatment insensitive malignant cells, possibly so-called cancer stem cells [138]. Patients with metastatic disease are treated with platinum plus etoposide combination therapy. Recently, several studies have demonstrated that immunotherapy such as anti-PD-L1 antibodies, in addition to the standard platinum plus etoposide regimen can improve the progression-free and OS in a small subset of these patients [139–141]. Despite the benefits brought by immunotherapies, SCLC still remains a largely lethal disease and there are no approved targeted therapies for SCLC. Therefore, a better understanding of the biology of SCLC is urgently required to identify new therapeutic vulnerabilities.

Lung cancer is the leading cause of cancer-related deaths worldwide, with a 5-year survival rate of <15% [98]. Lung cancer is a highly heterogenous disease that can be classified into four histopathological subtypes. Approximately 15% percent of lung cancer cases are classified as small cell lung cancer (SCLC). The remaining ~85% of cases are classified as non-small cell lung cancer (NSCLC), which can be further divided into adenocarcinoma (LUAD; ~40% of NSCLC), squamous cell carcinoma (LUSC, ~25% of NSCLC) and large cell carcinoma (~15% of NSCLC) [99].

i. Lung adenocarcinoma
  LUAD, the most prevalent subtype of NSCLC, is characterised by its glandular differentiation and is located most frequently in the periphery of the lung [100]. The genomic landscape of somatic alterations is distinct between different lung cancer subtypes, and a number of molecular subtypes have been identified in LUAD based on their oncogenic drivers [99]. For example, activating mutations of the gene encoding *epidermal growth factor receptor* (*EGFR*) or *KRAS*, or rearrangements of the genes encoding *anaplastic lymphoma kinase* (*ALK*) or *ROS proto-oncogene 1* (*ROS1*) have led to the oncogene-centric molecular classification of NSCLC [101]. Although there are known associations between subtypes of lung cancer with distinct oncogenic drivers and their histological appearance as well as growth patterns [102], tumours driven by the same oncogenic alterations can exhibit significant molecular and clinical heterogeneity [101]. This is in part due to the diversity of oncogenes that drive the different cases of lung cancer [103]. For example, *KRAS* mutations have been observed in various codons, with the majority of mutations occurring at codon 12, while mutations at codons 13, 10 and 61 are less frequent [103]. In NSCLC, the most common *KRAS* mutations include *KRAS*^*G12C*^ (39%), *KRAS*^*G12V*^ (18–21%) and *KRAS*^*G12D*^ (17–18%) [104]. The G12D mutation is more common in non-smoking NSCLC patients, whereas the G12C mutation is more prevalent in smoking NSCLC patients [105, 106]. These different amino acid substitutions exert distinct impact on the downstream mitogen-activated protein kinase (MAPK) signalling pathway, involving PI3K/AKT, mitogen-activated protein kinase kinase (MEKK) and RAS-like (RAL) GTPases. These are important factors that contribute to the overall survival (OS) and therapeutic response of lung cancer patients [103]. These subgroups of *KRAS*-mutant LUAD exhibit different responses to anti-cancer therapeutics and have distinct impact on the TME [101, 107]. There is emerging evidence that co-occurrence of specific mutations in oncogenes and tumour suppressor genes can contribute to the molecular and clinical heterogeneity in lung cancer [101]. For example, mutations of *p53*, mutations or loss of *liver kinase B1* (*LKB1*) (also known as *serine/threonine kinase 11* (*STK11*)), which is frequently associated with the loss of *Kelch-like ECH-associated protein 1* (*KEAP1*), and bi-allelic inactivation of *cyclin-dependent kinase inhibitor 2A* (*CDKN2A*) and *cyclin-dependent kinase inhibitor 2A* (*CDKN2B*) have been identified as three major co-occurring genetic alterations in *KRAS*-mutant LUAD. Like for many other cancers, mutations in p53 are common in lung cancer and certain hotspot mutants predominate. The most common p53 mutations in human LUAD occur at codon R273, while mutations at R175, which frequently occur in other cancer types, are less common in LUAD [108] (Fig. 3). In human NSCLC, conflicting results have been reported on the role of *p53* mutations as a prognostic marker [109–113], likely due to the differing functional effects of specific *p53* mutations. These findings highlight the importance of further categorising lung cancers with mutant *KRAS* and mutant *p53* based on their genotype, including particular mutations in *p53*.

## Insights into the role of mutant p53 in lung cancer from mouse models

Genetically engineered mouse models (GEMMs) enlighten the study of human cancers, as the gene modifications in GEMMs mimic the oncogenic mutations underpinning cancer formation in humans and enable the study of specific oncogenic driver events. Mutations of *KRAS* (32%) and *p53* (46%) are two of the most common gene mutations in LUAD and the co-occurrence of these two gene mutations is frequently observed [99]. In GEMMs of lung cancer, the *Kras*^*LSL-G12D/+*^ mouse model is the most widely used mutation for the study of *KRAS*-mutant LUAD. In this model a *lox-stop-lox* (LSL) cassette was inserted upstream of the transcriptional start site within a mutant *Kras*^*G12D*^ allele knocked into the endogenous *Kras* gene. Intra-nasal delivery of a CRE recombinase results in DNA recombination between the *loxP* sites, leading to the removal of the stop codon and consequently the expression of mutant *Kras* [142]. The expression of oncogenic KRAS^G12D^ protein in murine lung epithelial cells induces the formation of adenomas that recapitulate early-stage lesions observed in human LUAD [142]. The *Kras*^*LSL-G12D/+*^ mouse model of lung cancer with conditional inactivation of the *p53* allele was first generated by the Jacks Laboratory [89]. In addition to the modification of the *Kras* allele in a more aggressive model, exons 2–10 of the *p53* allele are also flanked by *loxP* sites, which results in the deletion of the *p53* allele when CRE is expressed [89]. Loss of *p53* cooperates with oncogenic *Kras* to induce LUAD with increased invasiveness and accelerates the progression from adenomas to adenocarcinomas [89]. Other studies using *Kras*-mutant LUAD mouse models with germline loss of *p53* have shown similar results [143]. However, as discussed above, most *p53* mutations in human cancers are missense mutations. It was found that mutant *p53* promotes disease progression of mutant *Kras*-driven lung tumours, and certain point mutations have been reported to contribute to tumorigenesis through DNE and/or GOF effects [89]. A previous study comparing the conditional mutations of *p53*^*LSL-R270H*^, *p53*^*LSL-R172H*^ and the *p53*^*fl/fl*^ allele in the *Kras*^*LSL-G12D/+*^ mouse model of LUAD revealed that while for all alterations the LOF effects were sufficient to promote *Kras*-initiated LUAD and drive metastasis to regional lymph nodes and distant organ sites, such as kidney in a small number of mice, the transcriptional processes governed by p53 to elicit this metastatic phenotype is however poorly understood. Of note, *p53*^*R270H*^ but not *p53*^*R172H*^ also acted in a dominant-negative manner in promoting tumorigenesis when the other allele was WT for *p53*. Moreover, the expression of mutant p53 was found to contribute to tumour heterogeneity by promoting the development of sino-nasal adenocarcinomas, which was not observed in tumours with a p53-deficient state [89]. These *Kras/p53* compound mutant mouse models recapitulate several characteristics of advanced human LUAD that are not commonly observed in the *Kras*-mutant*/p53*-deficient mouse models. Despite all the discoveries made from studies using these mouse models, which demonstrate the importance of p53 in lung cancer, the mechanism by which p53 suppresses lung cancer development remains unclear. Recently, a study by Kaiser et al. comparing the *Kras*-mutant LUAD mouse models with *p53* knockout, WT *p53* or hyperactive *p53* alleles with enhanced tumour suppressor activity has revealed the role of p53 loss in the initiation and progression of LUAD. It was found that tumour suppression by p53 in this setting involves the repurposing of the role of p53 in tissue repair, whereby p53 promotes alveolar type 1 cell differentiation of transitional (i.e. early neoplastic) cancer cells. Inactivation of *p53* leads to the inappropriate persistence of transitional cancer cells thereby promoting progression of LUAD [144] (Table 1).

**Table 1:** Summary of *p53* mutant lung cancer mouse models.

Cancer type	Genotypes	Features
Lung adenomas	*Kras*^*LSL-G12D/+*^ [142]	Recapitulates early-stage lesions observed in human LUAD [142]
LUAD	*Kras*^*LSL-G12D/+*^*;p53*^*fl/fl*^ [89]	Increased invasiveness and accelerated progression from adenomas to adenocarcinomas [89, 143]
	*Kras*^*LA1*^*;p53*^*-/-*^ [143]	
	*Kras*^*LSL-G12D/+*^*;p53*^*LSL-R172H*^ [89]	LOF effects in promoting *Kras*-initiated LUAD. Tissue-specific GOF effects in promoting sino-nasal adenocarcinomas [89]
	*Kras*^*LSL-G12D/+*^*;p53*^*LSL-R270H*^ [89]	LOF effects in promoting *Kras*-initiated LUAD. Tissue-specific GOF effects in promoting sino-nasal adenocarcinomas [89]
		DNE in promoting sino-nasal adenocarcinomas [89]
	*Kras*^*LSL-G12D/+*^*;p53*^*53/54,53/54*^ [144]	Tumour suppression by p53 involves the repurposing of the role of p53 in tissue repair [144]
	*Kras*^*LA2/+*^;*p53*^*LSL/LSL*^;*Rosa26*^*CreERT2*^ [151]	Restoration of WT p53 expression eliminates high-grade tumours [151, 154]
	*Kras*^*LSL-G12D/+*^*;p53ER*^*TAM*^ [154]	
SCLC	*Rb*^*F19/F19*^*;p53*^*F2-10/F2-10*^ [145]	Induces aggressive neuroendocrine lung tumours [145]
	*p53*^*flfll*^*;Rb1*^*fl/fl*^*;Pten*^*fl/fl*^ [146]	Exhibits variant characteristics of SCLC as well as large cell neuroendocrine carcinoma and NSCLC [146]
	*Rb1*^*fl/fl*^;*p53*^*fl/fl*^;*Rbl2*^*fl/fl*^ [148]	Shares similar features with the *p53*/*Rbl1* double knockout mouse model. Reliably recapitulates the ASCL1-high/NEUROD1-low subtype of human SCLC [148]
	*Rb1*^*fl/fl*^*;p53*^*fl/fl*^*;Myc*^*LSL/LSL*^ [149]	Representative of the variant subtype of human SCLC. Displays a high level of plasticity [149]
	*Rb1*^*fl/fl*^*;p53*^*XTR/XTR*^*;Rbl2*^*fl/fl*^ [157]	Restoration of the expression of WT p53 limits the growth and metastasis of autochthonous SCLC [157]

Numerous GEMMs have been developed to study SCLC, with almost all of them containing conditional alleles of *Rb1* (floxed exon 19) and *p53* (floxed exons 2–10). In these mice aggressive neuroendocrine lung tumours can be induced upon intra-nasal or intra-tracheal delivery of Adeno-Cre viruses [145]. Additional deletion of *Pten* or *Rbl2* and stabilisation/over-expression of MYC have been shown to further accelerate tumorigenesis and promote metastasis in these SCLC mouse models initiated by the loss of *Rb1* and *p53* [146–148]. For example, the *p53/Rbl1/Pten* triple knockout mouse model exhibits variant characteristics of SCLC as well as large cell neuroendocrine carcinoma and NSCLC [146]. Conversely, the *p53*/*Rb1*/*Rbl2* triple knockout mouse model shares similar features with the *p53*/*Rb1* double knockout model, and they both reliably recapitulate the ASCL1-high/NEUROD1-low subtype of human SCLC [148]. In contrast, the *p53*/*Rb1* double knockout model with Cre-activated mutant *Myc*^*T58A*^ that leads to stabilised MYC proteins, is representative of the variant subtype of human SCLC [149]. Tumour cells with this genotype display a high level of plasticity, with an ASCL1-high/NEUROD1-low phenotype at early-stage disease and a NEUROD1-high/ASCL1-low phenotype in invasive late-stage tumours [148] (Table 1). These observations suggest a possible plasticity between ASCL1 and NEUROD1 tumour cell phenotypes and implicate MYC as a driver of this process.

It was hypothesised that when WT *p53* expression is introduced into tumour cells, the WT p53 proteins will be activated and stabilised due to stress stimuli, such as oncogene expression, hyperproliferative signals and DNA damage. This would then lead to activation of p53 target genes and the subsequent induction of apoptosis or senescence of tumour cells [150]. This hypothesis has been tested in GEMMs of various cancers including lymphoma, lung cancer and liver carcinoma [151–154]. In a WT *p53* restorable mouse model of LUAD, the *LSL-*WT *p53* allele in the mice is phenotypically identical to the knockout state of the allele, but the expression of WT *p53* can be induced upon the removal of LSL by a CRE recombinase [151]. Using this mouse model in concert with activation of oncogenic Kras^G12D^, it was found that the induced expression of WT p53 eliminates high-grade tumours but not low-grade adenoma lesions [154–156]. Similar results were observed in other NSCLC mouse models containing *LSL-Kras*^*G12D*^ and *p53ER*^*TAM*^ alleles, whereby *p53* can be switched between the knockout and the WT state by tamoxifen administration [154]. In SCLC, the *Rb1*^*fl/fl*^*;p53*^*XTR/XTR*^*;Rbl2*^*fl/fl*^ mouse model was used to assess the effects of p53 restoration on tumour expansion. In this model, WT *p53* can be inactivated by a CRE recombinase and then restored by a FlpO recombinase. It was found that p53 restoration limited the growth and metastasis of autochthonous SCLC. Intertumoral heterogeneity was observed between SCLC tumour subtypes in response to the restoration of WT p53, such that this led to cell senescence in a subset of tumour cells but non-apoptotic cell death in other subsets of tumour cells [157] (Table 1).

Overall, GEMMs provide valuable insights into the molecular mechanisms driving cancer formation and development. Studies using the *p53* restorable mouse models suggest that reactivation of WT p53 might be a promising therapeutic strategy for treating mutant *p53*-driven lung cancers. The use of GEMMs facilitates our understanding of lung cancer biology, as well as the development of new therapeutic interventions.

## The prognostic role of p53 mutations

While *p53* mutations are found in over half of NSCLC [158, 159] and over 75% of SCLC [133, 134], the impact of *p53* mutations on prognosis remains controversial and can vary between lung cancer subtypes and tumour stage. Several studies have shown that LUAD patients with *p53* mutations have worse OS compared to patients with WT *p53* lung cancers, whereas no significant differences were observed in LUSC patients [160–162]. Other studies focusing on tumour stage have reported that mutations in *p53* are associated with worse OS in stage I NSCLC patients but not in stage II and stage III patients [110, 112]. Of note, the differences in therapeutic histories of patients and the small number of patients included in some of these studies could be confounding factors that led to contradictory results. In SCLC, it was found that *p53* mutations provided tumour cells with a growth advantage in metastatic disease and growth in tissue cultivation [134, 163]. Consistent with this finding, other studies have reported that the proportion of tumours with mutant *p53* is higher than those with WT *p53* in late-stage SCLC patients [164]. Furthermore, histological staining of mutant p53 proteins in over 100 SCLC tumour specimens revealed that the presence of mutant p53 is associated with poor patient survival, supporting the role of mutant p53 as a predictive marker of poor prognosis in SCLC [165].

While most studies only distinguished between cancer patients with WT *p53* versus mutant *p53*, the diverse spectrum of *p53* mutations was found to correlate with the heterogeneous clinical outcomes. Poeta et al. proposed to categorise *p53* mutations according to their effects on protein function. They classified p53 mutations into two groups: disruptive mutations which can lead to the LOF of the mutant p53 proteins, and non-disruptive mutations which partially retain some functions of WT p53 and are reportedly often associated with DNE and alleged GOF effects of mutant p53 [166]. Studies following these criteria have shown that non-disruptive *p53* mutations are associated with significantly shorter OS in advanced NSCLC patients, whereas *p53* mutations, as a whole, showed no significant correlation with OS. This may be explained by the possible DNE and GOF effects of non-disruptive mutant p53 proteins that lead to increased proliferation, metastasis and chemo-resistance, whereas disruptive p53 mutations are unlikely to exert DNE and GOF effects [167]. Different impacts of mutant *p53* on patient outcome were also observed in other cancers, including head and neck cancer, chronic lymphocytic leukaemia (CLL) and breast cancer [166, 168, 169]. However, some recent studies provided evidence that for the sustained expansion of cancers, alleged GOF effects of mutant p53 proteins are not critical [170–172].

## Immune and metabolic microenvironments in *p53* mutant tumours

It is widely accepted that tumour progression is not only impacted by cell autonomous features but also by the TME. Mutant p53 proteins have been found to promote a pro-oncogenic TME by modulating the secretion of cytokines and chemokines by the tumour cells [173]. In addition, p53 also plays an important role in anti-tumour immunity through the regulation of innate and adaptive immune profiles [174] and/or the expression of immune-inhibitory checkpoint regulators by the cancer cells [175].

CD4^+^ helper T cells, CD8^+^ cytotoxic T cells, NK cells and in certain cases, neutrophils contribute to immune surveillance of tumour cells [176–179], whereas other immune cells, such as regulatory T cells and certain myeloid cell subsets, were reported to suppress anti-tumour immune responses [180–182]. Tumour-associated macrophages (TAMs) are a major component of the TME. Two main polarisations of TAMs include M1-like macrophages, which exhibit anti-tumour activities by stimulating the adaptive immune response and inflammation and, conversely, M2-like macrophages, which exhibit tumour promoting activities by suppressing the immune response in the TME [183]. In lung cancer, TAMs are the most abundant immune cells present in the TME [184], and they were reported to promote the proliferation, epithelial–mesenchymal transition and metastasis of cancer cells, which results in poor patient outcomes [185, 186]. A significant increase in TAMs was observed in NSCLC and other cancers in response to loss of p53 [187]. NSCLC cells were found to facilitate the polarisation of TAMs towards the M2 phenotype in vitro [185]. However, other studies showed an increase in M1-like macrophages in *p53* mutant LUAD and LUSC patients [188, 189]. Furthermore, mutant p53 expressed in cancer cells has been reported to impact macrophage behaviour through its GOF effects thereby supporting tumour development [190]. T cell-mediated killing of tumour cells is critical for anti-tumour immunity. The activity of T cells during an immune response is regulated by the expression of immune checkpoint regulators, such as PD-1 [191]. Tumour cells exploit this pathway by upregulating the expression of ligands of these regulators, such as PD-L1, to dampen the immune response. The loss of *p53* in malignant cells is correlated with increased expression of PD-L1 in cells of the TME in many cancers, including lung cancer [174, 192, 193]. It has been reported that *p53* mutant lung cancer patients exhibited increased expression of immune checkpoint regulators, enhanced CD8 T cell infiltration and increased expression of effector T cell and interferon-γ associated genes [194]. This may indicate the predictive value of mutant *p53* for responses to ICB in lung cancer. Of note, there are also studies showing that in murine lung cancer models, p53-deficient tumour cells downregulated antigen presentation by reducing the expression of MHC-I, consequently exhibiting poorer responses to ICB compared to WT *p53* expressing tumours [195]. Concordant observations were found in human lung cancer, where NSCLC patients with *p53* truncating mutations had shorter OS with immunotherapy compared to patients with WT p53 expressing tumours [196]. Further research is required to better understand the clinical predictive value of the different *p53* mutations in lung cancer.

Metabolic reprogramming in the TME can result in significant changes in the function and response of the immune system. Recently, it was reported that glucose metabolism is associated with the phenotype of certain immune cells, such as macrophages, dendritic cells and CD8^+^ T cells [197–199]. In a study of 495 LUAD patients, genes involved in the glycolysis–gluconeogenesis metabolic pathways were shown to be correlated with *p53* mutations [188]. As a result of accelerated glycolysis in tumour cells, high levels of lactate are generated. The accumulation of lactate in the TME not only facilitates the progression and metastatic spread of tumour cells, but also exerts immune modulatory effects on tumour infiltrating immune cells. It was found that lactate suppresses the proliferation and cytokine production of cytotoxic T lymphocytes and thereby impairs their anti-tumour activity [200]. Furthermore, tumour cell derived lactate was shown to inhibit the differentiation and activation of dendritic cells and the secretion of TNF by monocytes [201, 202]. High levels of lactate were also found to enhance the production of IL-23, a cytokine that contributes to tumour-associated inflammation [203, 204]. Taken together, understanding the contributions of immune and metabolic parameters to tumour development is crucial for improving the predictive value and therapeutic strategies for *p53* mutant lung cancers.

## Targeting p53 for the treatment of lung cancer

Many anti-cancer therapeutics kill cancer cells by inducing DNA damage which triggers WT p53-mediated apoptosis and/or cell cycle arrest/cell senescence. Accordingly, loss or mutation of *p53* can contribute to the resistance of diverse tumour cells to cytotoxic drugs [6]. It has been reported that mutant p53 protein can negatively impact the cytotoxic effects of cisplatin treatment for NSCLC via the upregulation of NRF2, a transcription factor that was reported to contribute to the resistance of malignant cells to several anti-cancer drugs [205]. Furthermore, patients with WT p53 expressing lung cancers were observed to exhibit better responses to cisplatin-based therapies compared to patients with mutant p53 expressing lung cancers [206, 207]. Due to the high mutation rate of malignant cells with defects in the p53 pathway and the fact that mutant p53 proteins are only highly expressed in malignant tissues but absent in normal tissues, targeting mutant p53 itself has been proposed to be a promising strategy for improving the treatment of chemo- and/or radio-resistant tumours.

**Fig. 4:**
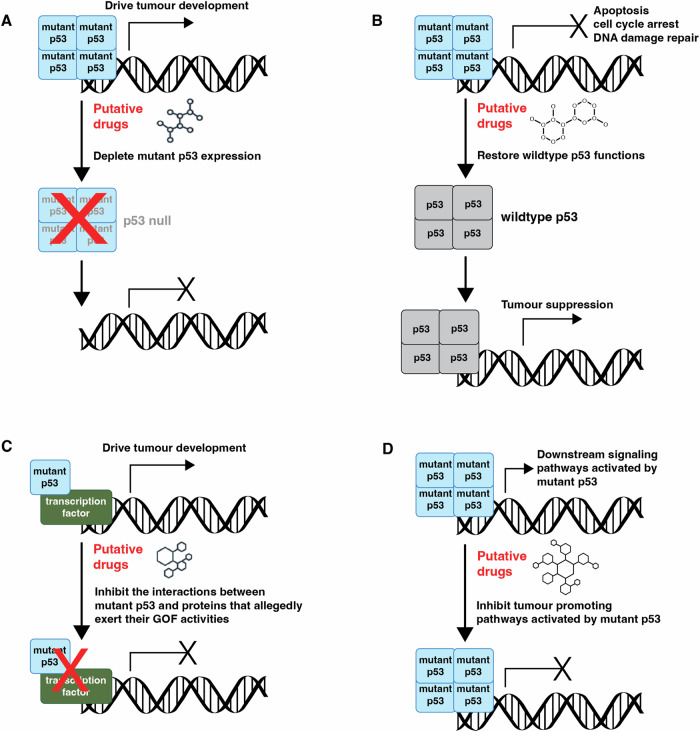
Therapeutic strategies to target mutant p53 in cancer. **A** Promoting the degradation of mutant p53 proteins through proteasomal or autophagy pathways. **B** Restoring WT p53 function in mutant p53 proteins in cancer cells. **C** Inhibiting the interactions between mutant p53 and proteins that allegedly exert their GOF activities. **D** Inhibiting downstream signalling pathways that may be activated by mutant p53.

Considerable efforts have been expended to explore strategies to therapeutically target mutant p53 in cancer cells. These strategies include promoting the degradation of mutant p53 proteins through proteasomal or autophagy pathways (Fig. 4A), restoring WT p53 function in mutant p53 proteins in cancer cells (Fig. 4B), inhibiting the interactions between mutant p53 and proteins that allegedly exert their GOF activities (Fig. 4C) as well as inhibiting downstream signalling pathways that may be activated by mutant p53 [208] (Fig. 4D). However, as stated above, recent studies have provided compelling evidence that removal of mutant p53 proteins had no impact on tumour expansion, metastasis and response to anti-cancer agents [170–172]. This demonstrates that any strategies aimed at removing or modulating mutant p53 proteins are unlikely to have therapeutic impact.

Many compounds have been developed to convert the mutant p53 protein into a WT p53 conformation, thereby restoring some WT p53 functions. For instance, PhiKan083 and PK7088 specifically target the mutant p53^Y220C^ protein by stabilising its structure, thereby increasing the levels of p53^Y220C^ proteins with WT conformation and activity [209, 210]. Other compounds targeting various mutant p53 proteins include PRIMA-1 and its methylated derivative APR-246, SCH29074 and CP-31398, which are thought to be able to restore WT TP53 function by promoting the proper folding of the mutant p53 protein [211–213]. Among all these compounds, APR-246 has been shown to effectively induce apoptosis in SCLC cell lines [214]. Furthermore, synergistic effects of APR-246 with cisplatin treatment were observed in both NSCLC and SCLC cell lines [215, 216], indicating its therapeutic potential in clinical settings. Importantly, although individual mutations and their associated GOF effects can result in variable therapeutic vulnerabilities, the restoration of WT p53 function exerts comparable effects across tumours with different mutant *p53* in lung cancer mouse models that can be switched from mutant *p53* to WT *p53* [217]. However, of note, recent studies have revealed that APR-246 does not actually kill malignant cells by targeting mutant p53 since CRISPR mediated removal of mutant p53 had no impact on sensitivity to this agent in a broad range of cancer cell lines [218].

The restoration of WT p53 function has the potential to improve the treatment outcome of lung cancer patients to standard therapy by enhancing the response of tumour cells to DNA damage-inducing agents [217]. However, studies using mouse models suggest that targeting mutant p53 might only be beneficial to patients with late-stage lung cancer, as mutant p53 proteins are only adequately activated in advanced tumours [154, 156]. Regardless, these findings provide valuable insights in the therapeutic impact of restoration of WT p53 function in lung cancer.

## Summary and future perspectives

p53 is a crucial tumour suppressor that protects cells from malignant transformation by activating a range of cellular responses, including apoptosis, adaptation of cellular metabolism, cell cycle arrest, cell senescence and DNA damage repair. *p53* is the most frequently mutated gene in human cancers and its mutations are associated with poor patient outcomes, although there is some controversy in lung cancer. Despite decades of research, the exact mechanisms by which mutant p53 proteins promote the initiation and sustained expansion of cancer remains a topic of extensive debate. Furthermore, the interplay between mutant p53 and other oncogenic drivers and the impact of co-mutations on the development and therapeutic responses of lung cancer are unclear. The development of GEMMs has allowed for insights into the biology of *p53* mutant cancers, as they reliably mimic the oncogenic mutations underpinning cancer formation in humans and hence enable the study of the impact of specific oncogenic driver events.

Although immunotherapy has achieved remarkable breakthrough in lung cancer treatment, patients with mutant p53 expressing lung cancers only show limited responses to immunotherapies. This highlights the urgent need for novel therapeutic approaches to enhance the efficacy of immunotherapies. *p53* mutations often confer resistance to many anti-cancer agents that induce DNA damage. Therefore, exploring strategies that specifically target mutant p53 are of great importance for improving the clinical outcomes for these cancer patients. Moreover, *p53* mutant lung cancers exhibit high levels of molecular and clinical heterogeneity, which leads to heterogeneous clinical outcomes. Therefore, characterisation of the impacts of different p53 mutant proteins is essential for improving the prognostic and predictive values of mutant p53 for treatment response, as well as for the development of personalised treatments for patients with *p53* mutant lung cancers. Overall, *p53* mutations remain an ongoing challenge in understanding the development of cancer as well as cancer therapy. Further research is required to address these pressing issues and advance our understanding of the many functions of WT p53 in tumour suppression and the impact of the different p53 mutant proteins in lung cancer.
